# Suppression of Methane Generation during Methanogenesis by Chemically Modified Humic Compounds

**DOI:** 10.3390/antiox9111140

**Published:** 2020-11-17

**Authors:** Elena Efremenko, Olga Senko, Nikolay Stepanov, Nikita Mareev, Alexander Volikov, Irina Perminova

**Affiliations:** 1 Faculty of Chemistry, Lomonosov Moscow State University, 119991 Moscow, Russia; senkoov@gmail.com (O.S.); na.stepanov@gmail.com (N.S.); mr.n.mareev@yandex.ru (N.M.); ab.volikov@gmail.com (A.V.); iperminova@gmail.com (I.P.); 2 N.M. Emanuel Institute of Biochemical Physics RAS, 119334 Moscow, Russia

**Keywords:** methanogenesis, quinones, humic acids, bioluminescence, antioxidant activity, anaerobic consortia, immobilization

## Abstract

The introduction of various concentrations of chemically modified humic compounds (HC) with different redox characteristics into the media with free and immobilized anaerobic consortia accumulating landfill gases was studied as approach to their functioning management. For this purpose, quinone (hydroquinone, naphthoquinone or methylhydroquinone) derivatives of HC were synthesized, which made it possible to vary the redox and antioxidant properties of HC as terminal electron acceptors in methanogenic systems. The highest acceptor properties were obtained with potassium humate modified by naphthoquinone. To control possible negative effect of HC on the cells of natural methanogenic consortia, different bioluminescent analytical methods were used. The addition of HC derivatives, enriched with quinonones, to nutrient media at concentrations above 1 g/L decreased the energetic status of cells and the efficiency of the methanogenesis. For the first time, the significant decrease in accumulation of biogas was reached as effect of synthetic HC derivatives, whereas both notable change of biogas composition towards increase in the CO_2_ content and decrease in CH_4_ were revealed. Thus, modification with quinones makes it possible to obtain low-potential HC derivatives with strongly pronounced acceptor properties, promising for inhibition of biogas synthesis by methanogenic communities.

## 1. Introduction

One of the serious modern environmental problems is the constant increase in the concentration of CH_4_ in the atmosphere due to the existence of landfills used for solid household waste accumulation [1], which significantly worsen the ecological state of the environment and negatively affect human health [2,3]. Organic matter in waste in such landfills is usually decomposed by anaerobic microbial consortia and converted into landfill gas, consisting mostly of methane (55–60% *v*/*v*) and CO_2_ (40–45% *v*/*v*), which forms a threat for possible ignition and subsequent smoldering of waste [4,5]. The need to reduce methane emissions from such landfills is today one of the urgent tasks of the world economy and ecology [6].

Previously, it was believed that global warming would cause a significant increase in methane emissions from vast wetlands into the atmosphere as a result of melting permafrost. However, the presence of humic compounds (HC) in these territories, as it turned out, can inhibit the release of CH_4_ [7,8]. This effect is associated today with the fact that HC act as a terminal electron acceptor (TAE) in anaerobic media [9]. It is believed that HC electron transfer in anaerobic systems competitively suppresses the reduction of other TAEs, including CO_2_, under conditions of methanogenesis [10].

To date, there are a number of studies devoted to the decrease under the influence of HC in the efficiency of methanogenesis, catalyzed by various anaerobic methanogenic consortia [11,12,13,14,15]. Using four different methanogenic consortia as an example, we also have previously revealed that the introduction of natural humic acids (HA) into the media at a concentration of up to 10 g/L leads to a decrease in the general energy status of microbial cells, which indicates the inhibition of their metabolic processes. It was found that in the presence of HA, regardless of the composition of natural methanogenic consortia, the composition of the resulting biogas can be slightly changed [16]. However, it was noted that the donor/acceptor capacity of HC themselves, extracted from natural sources, varies greatly due to differences in their chemical composition [17,18,19]. In particular, when studying the donor/acceptor capacities of 15 samples of HC isolated from water and terrestrial samples, it was found that aqueous HC had higher donor and lower acceptor capacities than terrestrial HC with comparable aromaticity. These differences were explained by the presence of a larger number of electron-donor phenolic fragments in the molecules of aqueous HC. The known study of antioxidant activity (AOA) of 18 samples of HC isolated from 9 different peat samples showed that all samples possessed AOA [20]. At the same time, HC with high AOA were characterized by higher aromaticity due to the significant content of phenolic, alcohol and ether groups. Taking this information into account, it becomes obvious for us that an additional directed chemical modification of HC can change their initial redox properties in a targeted way. Based on the difference in AOA, it is possible to estimate the number of oxidized groups in the HC structure and thereby increase the maximum acceptor capacity of HC preparations, which can be effective in inhibiting of landfill methanogenesis. The idea on special introduction of electron-withdrawing quinone fragments to the structure of terrestrial HC looked very attractive. In this regard, it seems advisable to use HC chemically modified with various quinone residues, which change the electron-withdrawing properties of HA by analogy with some natural variants. It is believed that quinone fragments in HC, in contrast to other TAEs, that are capable of accepting electrons during the hydrolysis of a large pool of organic substances, which leads to a stable high level of CO_2_ accumulation, are oxidized during hydrogenation reactions, which leads to the restoration of their ability to accept electrons [21]. Carrying out targeted synthetic modification of HC, it seemed possible to take a step towards environmentally friendly and affordable reagents that make it possible to effectively control the accumulation of methane in situ.

The study of the possible efficiency of using such synthetic derivatives of HC, as well as their concentration, which can provide the desired result and inhibit methanogenesis, is of great scientific and practical importance. However, the potential practical use of such chemically modified HC requires a careful assessment of their toxicity and safety for the environment. Bioluminescent analytical methods can be used to control metabolic effects, which are the response of microbial cells to changes occurring with them and effects associated with the possible toxic influence of the substances used on cells. Among such bioluminescent methods is the determination of the intracellular concentration of adenosine triphosphate (ATP) using firefly luciferase, since ATP is an indicator of cell viability and their metabolic activity [22,23,24,25]. At the same time, firefly luciferase itself can also be used as an identifier of compounds capable of reducing the catalytic activity of enzymes involved in various biological processes, since it is known to be inhibited by a number of substances [26,27]. *Photobacterial* cells, which bioluminescence intensity is actively used to assess the toxicity of various substances [28], can also be successfully used to assess the biosafety of new chemically synthesized and modified compounds [29]. It seems that in the case of the search and development of substances used to regulate the metabolic processes of any microorganisms, the use of these two bioluminescent methods (especially in combination) should provide a prompt and adequate assessment of the characteristics of chemically modified HC. It is known that the cells of methanogenic consortia are self-immobilized into granules under natural conditions. That contributes to the maintenance of anaerobiosis and an increase in the stability of the functioning of microorganisms within such self-organized microbial communities [30]. The cells of methanogenic consortia, artificially immobilized in a macroporous cryogel of polyvinyl alcohol (PVA), in their physiological state and metabolic activity are close to natural self-immobilized methanogenic associations [25] and therefore they are interesting to be used as a model of real biosystems in studying the effect of new HC derivatives on consortia and methanogenesis.

The aim of this work was to study the effect of specially chemically synthesized quinone derivatives of HC using hydroquinone (HQ), 2-methylhydroquinone (MeHQ), 2-hydroxy-1,4-naphtoquinone (NQ) on the characteristics of the methanogenesis process realized under the action of various anaerobic consortia, including them in an immobilized form, as well as assessment of the toxicity of artificially created HC derivatives using bioluminescent methods.

## 2. Materials and Methods

### 2.1. The Source of the HC, Their Modification, Redox Capacity and Antioxidant Capacity

**Figure 1:**
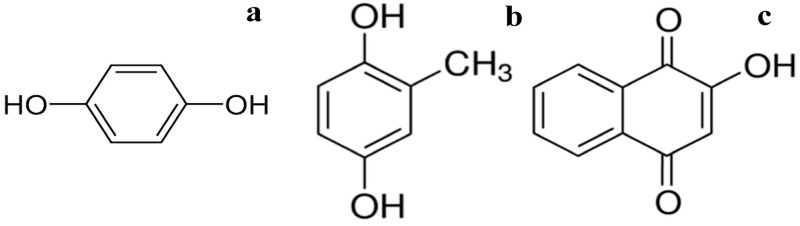
Quinone centers incorporated into the humic compounds (HC) used in this study: HQ (**a**), MeHQ (**b**), NQ (**c**).

In this study HC (potassium humate (PH) from leonardite and fulvic acids (FA) of groundwater (Humintech GmbH, Grevenbroich, Germany)) were used. Samples of quinonoid-enriched HC with certain centers used for modification (Figure 1) were obtained by Fenton reagent (Fe^2+^, H_2_O_2_) treatment under alkaline conditions [31].

Potassium ferricyanide was used to determine the redox capacity of HC [32]. Determination of the antioxidant activity of HC samples was defined as Trolox equivalent antioxidant capacity (TEAC) using a known method [33]. The reduced forms of HC were obtained using a 0.5% NaBH_4_ solution. For this, 5 mL of a 0.02% HC solution was added to 5 mL of NaBH_4_ solution, the mixture was exposed for 24 h, after which the excess of NaBH_4_ was removed by adding 1 M HCl to pH 7.0 ± 0.1.

### 2.2. Anaerobic Consortia and Their Immobilization

Three anaerobic consortia were used in this work: the 1st one was natural anaerobic consortium isolated from the Kashira landfill (Kashira, Russia) [34]; the 2nd—anaerobic consortium obtained from a digester which treated cattle waste (Dmitrov, Russia); the 3rd—anaerobic consortium obtained from the wastewater of a food plant (Kashira, Russia). The main characteristics and storage conditions of used consortia were previously described in detail [16]. The samples of anaerobic sludge were immobilized into PVA (poly(vinyl alcohol) cryogel according to the previously developed technique [35].

### 2.3. Bioluminescent Tests

The immobilized cells of *Photobacterium phosphoreum* B-1717 (All-Russian Collection of Industrial Microorganisms) were used for bioluminescent test. The test procedure was described previously [36]. The residual intensity of bioluminescence was analyzed after the exposure of the cells to a certain HC taken in concentration from range 0.1–10 g/L for 0.5 h after its addition to medium.

The intracellular ATP concentration in samples of anaerobic consortia was analyzed using the bioluminescent luciferin–luciferase method [16].

### 2.4. Methanogenic Tests

The three consortia used in the work were tested in media with different concentrations of HC using 100 mL vials applied for anaerobic research (Sigma-Aldrich, St. Louis, MO, USA). The culture medium was prepared on the basis of 0.1 M phosphate buffer (pH 7.2) and contained 1 g COD (chemical oxygen demand)/L, while glucose was used as a carbon source at 37 ± 1 °C. Vials with the medium were purged with helium for 0, 5 h to remove dissolved air. The inoculum to substrate ratio (both expressed in g VS/L) was 3.5 ± 0.1. The total volume of medium with consortium inoculum was 50 mL. The efficiency of methanogenesis was calculated as describe previously [16].

### 2.5. Analytical Methods

The content of H_2_, CH_4_ and CO_2_ in the gas phase was controlled using a Crystallux-4000M gas chromatograph (RPC (Research and Production Company) “Meta-chrom”, Yoshkar-Ola, Russia) equipped with a PAROPAK QS column. The column had a thermal conductivity detector that was operated at 50 °C. Argon was used as a carrier gas at a flow rate of 30 cm^3^/min. Gas samples (1 cm^3^) were taken by syringe and injected to the analytical column. All measurements were performed in triplicate and data was analyzed using NetChrom software (RPC “Meta-chrom”, Yoshkar-Ola, Russia).

The data are presented as means of at least three independent experiments ± standard deviation (± SD). Statistical analysis was performed using SigmaPlot (ver. 12.5, Systat Software Inc., San Jose, CA, USA).

## 3. Results

### 3.1. Analysis of Redox-Capacity and Antioxidant Activity of Various HC Chemical Derivatives

The determination of redox capacity of different quinone-enriched and natural HC was undertaken (Table 1). Since it was previously shown that the introduction of PH into the medium leads to a noticeable inhibition of methanogenesis for different anaerobic consortia, while the introduction of FA (fulvic acids) into similar media, on the contrary, stimulates methanogenesis in media with a half of the tested consortia [16], we decided to focus on the study of HA derivatives.

**Figure 2:**
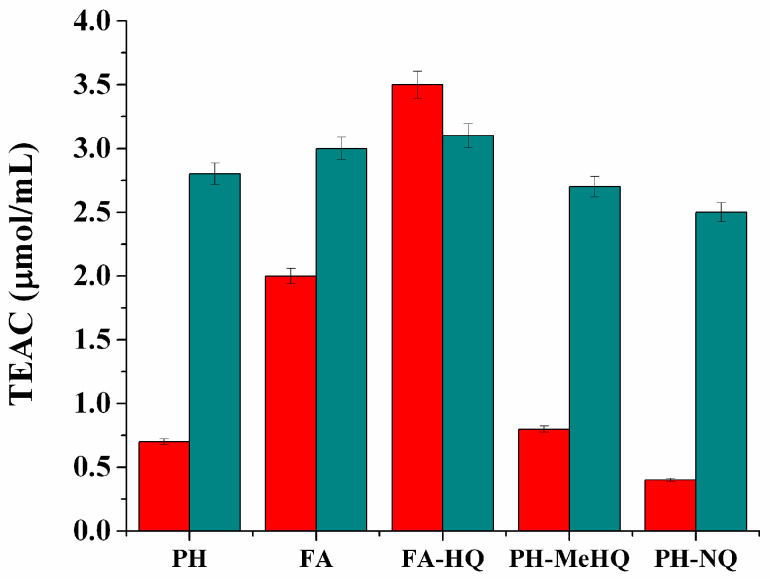
**TEAC** (Trolox equivalent antioxidant capacity) of HC and their quinone derivatives before NaBH_4_ treatment (red columns) and after it (blue columns). **PH**: potassium humate. **FA**: fulvic acids. **FA-HQ**: fulvic acids after modification with hydroquinone. **PH-MeHQ**: potassium humate after modification with 2-methylhydroquinone. **PH-NQ**: potassium humate after modification with2-hydroxy-1,4-naphtoquinone.

According to the data obtained, the natural FA had the maximum reducing capacity, while the same parameter of natural PH was low. The reducing capacity of FA after modification with hydroquinone (FA-HQ) remained rather high, while the reducing ability of the derivatives of PH obtained with naphthoquinone (PH-NQ) and methylhydroquinone (PH-MeHQ) was the lowest. It indicated the high acceptor properties of the PH derivatives obtained. The difference between TEAC (Trolox equivalent antioxidant capacity) values, which were established for different HC samples before and after NaBH_4_ reduction, is reflected in Figure 2.

According to the results obtained, TEAC of samples of PH and its quinone derivatives were noticeably lower than that of FA, which can be explained by the presence of a greater number of phenolic groups in the FA structure. However, the TEAC values for different HC samples leveled off and were on the order of 2.5–3.0 μmol/mg after treatment with NaBH_4_.

### 3.2. Influence of Modified HC on the Bioluminescence Intensity of Immobilized P. phosphoreum B-1717 and Luciferase

**Figure 3:**
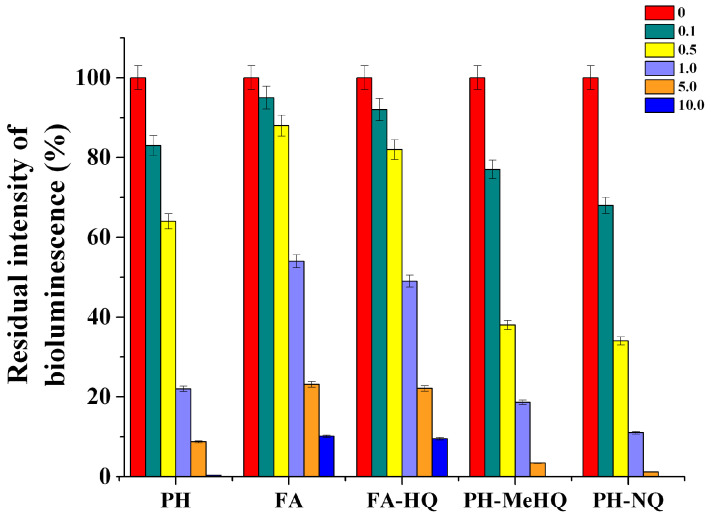
The residual bioluminescence intensity of immobilized *P. phosphoreum B-1717* cells in presence of various concentrations (g/L) of different types of HC. Basically the difference in the mean values among the treatment groups was statistically significant according one-way ANOVA (one-way analysis of variance) (*p* < 0.01).

Using immobilized cells of photobacteria, the toxicity of the obtained chemically modified HC samples was evaluated. It was found that there is a correlation between the concentration of HC samples introduced into the medium with *P. phosphoreum* B-1717 cells and the residual level of bioluminescence of immobilized *P. phosphoreum* B-1717 cells (Figure 3). Thus, with an increase in the concentration of the studied HC samples in a medium with *P. phosphoreum* B-1717, the intensity of their bioluminescence decreased. Pearson correlation coefficient between the concentration of HC samples, introduced into the medium with *P. phosphoreum* B-1717 cells and the residual level of bioluminescence was estimated. Pearson correlation coefficient for all HC samples was below −0.7, that testifies to the functional linear feedback.

It is obvious that the FA samples had the minimum inhibitory effect on the bioluminescence of *P. phosphoreum* B-1717 and the PH-NQ and PH-MeHQ samples had the most toxic effect on the cells of the *P. phosphoreum* B-1717. The intensity of *P. phosphoreum* B-1717 cells bioluminescence decreased up to 100% when concentration of modified HC (PH-NQ and PH-MeHQ) in medium was more than 5 g/L. In comparison with these results, in the case of FA preparations, even at a concentration of 10 g/L, the intensity of residual bioluminescence of immobilized *P. phosphoreum* B-1717 cells was about 10 ± 1%. Testing of different HC at various concentration with one-way ANOVA (one-way analysis of variance) reveals only two pairs with statistically non-significant differences were obtained: PH-NQ and PH-MeHQ both taken at 10 g/L (*p* > 0.19). Difference of all other pairs was statistically significant (*p* < 0.01, Table S1).

These results suggest that these chemically synthesized HC derivatives can provide the most effective suppression of cell metabolic activities of microorganisms that are part of microbial consortia in the methanogenesis, in order to reduce CH_4_ emission. Taking into account these results, it can also be assumed that the introduction of HC at a concentration above 1 g/L into media with anaerobic methanogenic consortia of microorganisms can be used to suppress their main metabolic processes.

**Figure 4:**
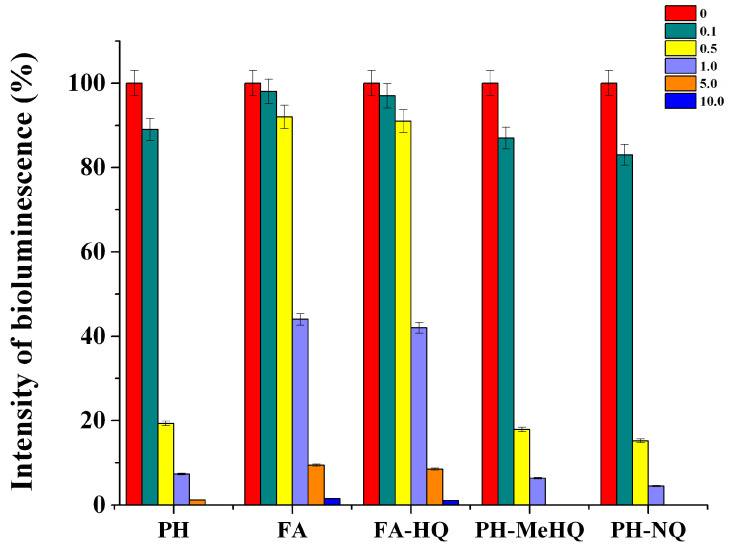
Influence of various HC and their concentrations (g/L) on the intensity of recorded bioluminescence when they are introduced into the reaction medium with luciferin-luciferase reagent and standard ATP samples. Basically the difference in the mean values among the treatment groups was statistically significant according one-way ANOVA (*p* < 0.05).

To analyze the effect of chemically modified HC on the metabolic characteristics of cells according to the level of intracellular ATP concentration, it was necessary to make sure that these substances cannot inhibit firefly luciferase used for determining ATP in microbial cells. In this regard, the influence of various samples of modified HC and their concentrations on the activity of this enzyme was evaluated (Figure 4).

It was found that, even at a HC concentration above 0.1 g/L, they showed an inhibitory effect on the luciferase reaction. At this concentration, PH-NQ and PH-MeHQ reduced the intensity of recorded bioluminescence by 13 and 17%, respectively, while FA samples decreased it by only 2–3%. With an increase in the concentration of HC in the medium, the intensity of bioluminescence decreased. When the concentration of preparations of potassium humate and its quinone-modified derivatives exceeded 1 g/L, the luminescence intensity was below 10%. Statistically non-significant differences were obtained with all HC samples at 10 g/L (*p* > 0.5). Difference of all other pairs was statistically significant (*p* < 0.05).

As in the case of *P. phosphoreum* B-1717 cells, the greatest inhibitory effect was revealed for PH and its modified derivatives. At the same time, the FA-HQ sample reduced the luciferase activity by more than 2 times only at a concentration of 10 g/L. In this regard, the necessity of preliminary dilution of those cell samples in which the concentration of ATP was further determined in the presence of high concentrations of different HC was taken into account.

### 3.3. Influence of Modified HC on the ATP Level of Cells in the Natural Anaerobic Consortia Catalyzing Methanogenesis

**Figure 5:**
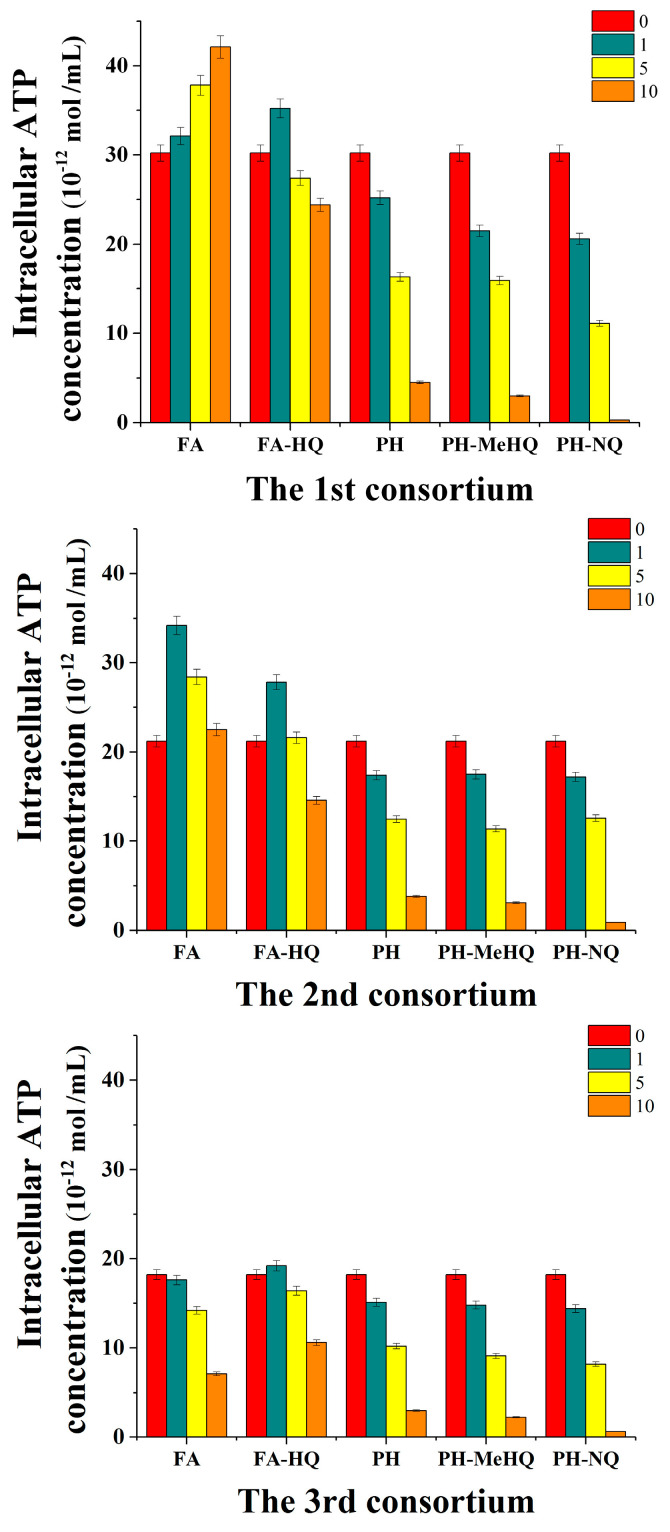
Influence of different concentrations of HC (g/L) on the intracellular ATP concentration in cell samples after anaerobic cultivation of methanogenic consortia.

Further, to assess the effect of modified HC on cell metabolism, the concentration of intracellular ATP was determined by the bioluminescent luciferin-luciferase method. It was realized for all tried consortia after methanogenesis (Figure 5). It was found that the introduction of HA quinone derivatives into nutrient media at concentrations above 1 g/L led to a decrease in the intracellular concentration of ATP in the cells of all anaerobic consortia. Moreover, this effect was enhanced with an increase in the concentration of HC in the studied concentration range (from 1 to 10 g/L). The inhibitory effect of PH-NQ on the cells of natural consortia was more pronounced than PH-MeHQ.

Thus, the presence of PH-NQ at a concentration of 10 g/L in culture media with cells of all three types of consortia resulted in a decrease in the concentration of intracellular ATP by more than 20 times. At the same time, when PH-MeHQ was introduced into the system at similar concentrations, the intracellular concentration of ATP decreased by 8–10 times.

The presence of the FA-HQ preparation in the nutrient medium did not have such a significant negative effect on the energy status of bacteria in methanogenic consortia as the modified PH preparations. And at a concentration of 1 g/L FA-HQ in the medium, the content of intracellular ATP in the cells of all anaerobic consortia even increased in comparison with the control values of this parameter. As the FA-HQ in the medium increased to 10 g/L, a slight inhibitory effect on the metabolic activity of cells was observed (20–40%).

### 3.4. Analysis of the Biogas Production Efficiency and Biogas Content Accumulated by Methanogenic Consortia in the Presence of Different Concentrations of Modified HC

**Figure 6:**
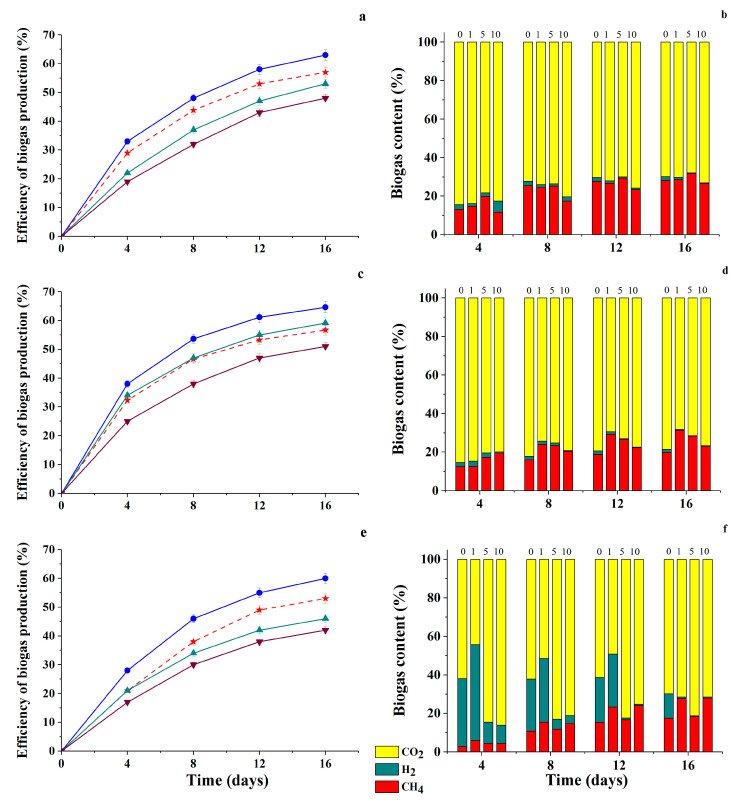
Efficiency of biogas production and biogas content during anaerobic cultivation of natural anaerobic consortia—the 1st (**a**,**b**), 2nd (**c**,**d**), 3rd (**e**,**f**)—in media containing 1 g/L glucose and different concentrations of FA-HQ (g/L): 1—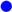, 5—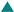; and 10—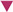, (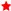
—control sample without HC in the medium). The theoretical maximal conversion of substrate to biogas was assumed as 100% efficiency of biogas production.

When studying the efficiency of biogas accumulation under the action of natural anaerobic consortia in the presence of different HC variants, it was found that the FA-HQ sample introduced into the nutrient medium at a concentration of 1 g/L stimulated the accumulation of biogas and an increase in its concentration to 10 g/L led to a slight decrease in biogas production in all tested consortia (Figure 6).

**Figure 7:**
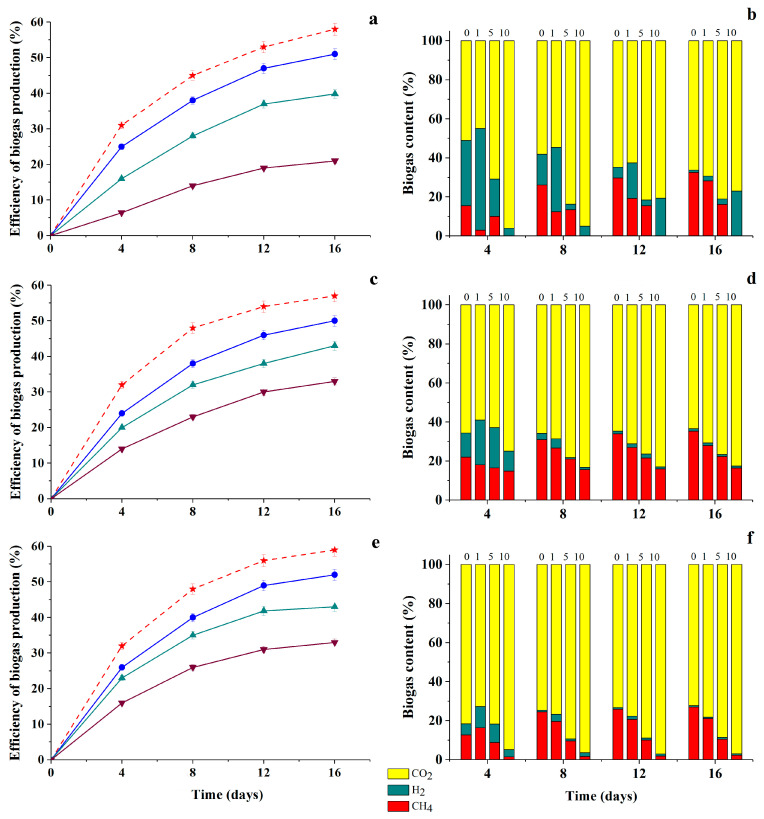
Efficiency of biogas production and biogas content during anaerobic cultivation of natural anaerobic consortia—the 1st (**a**,**b**), 2nd (**c**,**d**), 3rd (**e**,**f**)—in media containing 1 g COD/L glucose and different concentrations of PH-MeHQ (g/L): 1—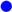, 5—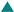; and 10—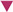, (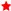
—control sample without HC in the medium).

**Figure 8:**
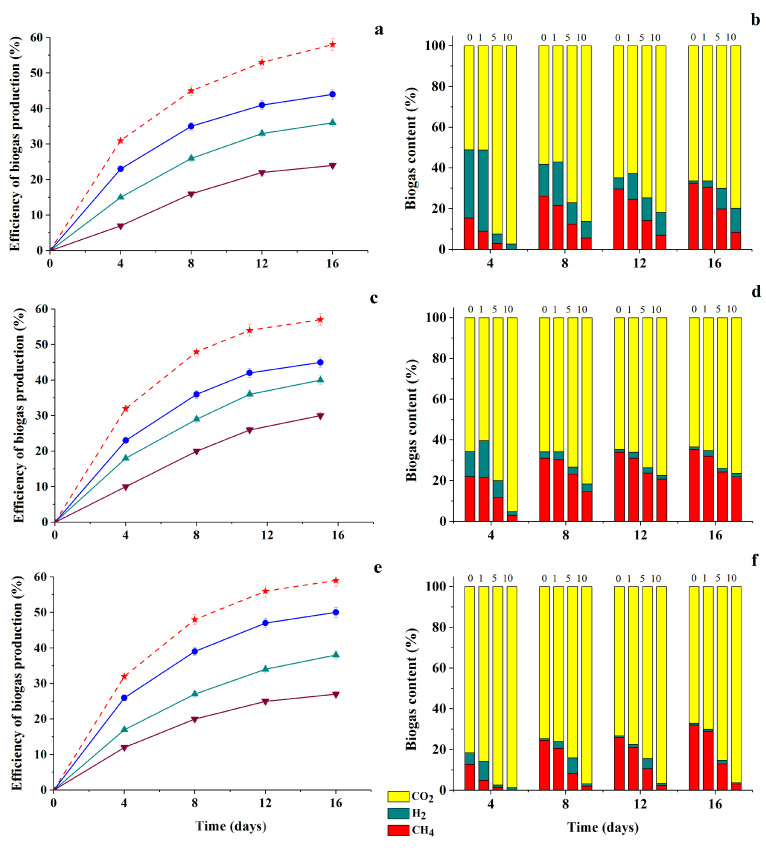
Efficiency of biogas production and biogas content during anaerobic cultivation of natural anaerobic consortia—the 1st (**a**,**b**), 2nd (**c**,**d**), 3rd (**e**,**f**)—in media containing 1 g/L glucose and different concentrations of PH-NQ (g/L): 1—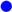, 5—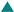; and 10—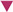, (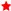
—control sample without HC in the medium).

Analysis of the composition of biogas accumulated as a result of methanogenesis in the presence of different concentrations of FA-HQ showed that the proportion of CH_4_ in the resulting biogas increased in comparison with the control (without FA-HQ) in the case of the 2nd consortium, regardless of the concentration of FA-HQ introduced into the medium (Figure 6d). A slight decrease in the proportion of methane at 10 g/L FA-HQ was noted in samples with the 1st consortium. And in the case of the 3rd consortium, an increase in the proportion of methane by more than 10% was observed at FA-HQ concentrations of 1 and 10 g/L. In general, the level of methane accumulation under the influence of various anaerobic consortia decreased with an increase in the initial concentration of modified HA preparations in culture media (Figure 7 and Figure 8). This clearly correlated with a decrease in the overall energy status of cells.

The lowest biogas production efficiency was obtained in samples with the 1st consortium, when PH-MeHQ was added to the medium and in samples with the 3rd consortium, when PH-NQ was added at a concentration of 10 g/L to the medium (Figure 7a and Figure 8e).

Analysis of the composition of biogas samples accumulated under the metabolic action of all three consortia showed a total decrease in the proportion of methane and an increase in the proportion of CO_2_ in its composition with an increase in the initial concentration of PH-MeHQ and PH-NQ up to 10 g/L (Figure 7b,d,f and Figure 8b,d,f). In this case, the introduction of 10 g/L PH-MeHQ into the nutrient medium in the case of the 1st consortium led to a complete cessation of CH_4_ synthesis (Figure 7b), that is, the efficiency of inhibition of methane formation in this case for the first time was 100%.

In a number of studied media variants, an increase in the proportion of hydrogen (20 times) in the accumulating biogas was noted in comparison with its amount in the control, that is, without the introduction of HC into the medium.

Undoubtedly, such a replacement of methane for hydrogen in biogas cannot be of practical interest, since the generated hydrogen can be converted by cells of anaerobic consortia into additional portions of methane after overcoming the inhibitory effect of HC and in addition, accumulated hydrogen poses a serious threat to the formation of an explosive situation in situ.

In particular, a high content of H_2_ remained in the composition of biogas formed during methanogenesis under the action of the 1st consortium in the presence of PH-NQ in the medium at concentrations of 5–10 g/L (Figure 8b). In all other samples, the proportion of hydrogen decreased by the end of the process (on the 16th day). Low methane content in biogas (3%) was also observed in samples from the 3rd consortium obtained in the presence of 10 g/L PH-NQ (Figure 8f).

### 3.5. Effect of Modified HC on Metahnogenesis Catalyzed by Immobilized Cells of Methanogenic Consortia

**Figure 9:**
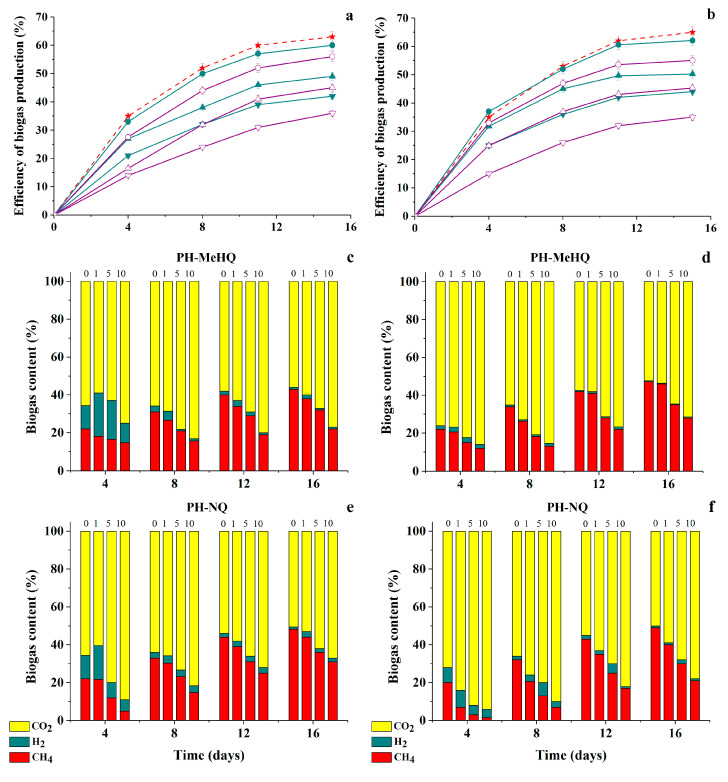
Efficiency of biogas accumulation and dynamics in its content as result of 16-days-cultivation of natural anaerobic consortia in immobilized form (**a**,**c**,**e**—the 1st consortium, **b**,**d**,**f**—the 3rd consortium) in the media containing 1 g/L glucose and various concentrations (g/L) of PH-MeHQ (filled cyan symbols) and PH-NQ (empty purple symbols): 1—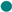, 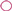 5—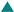, 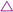 and 10—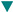, 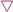. Control without HC—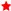 (dashed line) (**a**,**b**).

In order to simulate the state of natural consortia under the conditions of introduction of chemically modified HA samples into their environments, two consortia from among the previously tested ones (the 1st and 3rd ones) were artificially immobilized in PVA cryogel by the method of physical cell inclusion and the parameters of their functioning were investigated. The efficiency of biogas accumulation and its composition during the cultivation of immobilized cells with different concentrations of PH-MeHQ and PH-NQ preparations were analyzed (Figure 9).

It was found that the inhibitory effects of modified HA samples on the process of methanogenesis catalyzed by immobilized cells are less pronounced in comparison with suspended cells of consortia. Thus, the biogas yield was on average 5–10% higher in the case of immobilized samples at the same concentrations of HA derivatives. However, the general trends persisted: the efficiency of biogas accumulation under the influence of anaerobic consortia, both in suspension and in immobilized form, decreased with an increase in the initial concentration of modified HA preparations introduced to the nutrient medium. There was also a decrease in CH_4_ content within resulting biogas.

## 4. Discussion

Samples of natural FA were characterized by the maximum reducing ability and antioxidant activity, while in the samples of PH and its quinone derivatives, these characteristics were noticeably lower, which can be explained by the presence of a larger number of phenolic groups in the FA structure. If we pay attention to the difference in the TEAC values established for the initial and reduced forms of different HC, it becomes obvious that the maximum difference is characteristic of the initial samples and derivatives of PH, which indicates the highest portion of oxidized fragments in their structure, which predetermine the high acceptor capacity of these substances.

Thus, additional modification with quinones makes it possible to obtain low-potential HC derivatives with strongly pronounced acceptor properties, which are most promising from the point of view of their possible application for inhibition of biogas synthesis by methanogenic communities. The FA samples had the minimum inhibitory effect on the bioluminescence of *P. phosphoreum* B-1717 and the PH-NQ and PH-MeHQ samples possessed the most toxic effect on the cells of *P. phosphoreum* B-1717.

This coincided with the results obtained earlier in the study of the effect of natural FA samples on the functioning of other cells, in particular, of various methanogenic consortia, when minimal inhibitory effects of FA on their biogas synthesis were revealed [16]. Thus, it can be assumed that the effects that are observed for different HC on the cells of *P. phosphoreum* B-1717 correspond to those effects that can be observed on methanogenic associations.

Similar results were obtained when analyzing the effect of chemically modified HC on the inhibition of firefly luciferase, which is used in the composition of the reagent for determining the concentration of intracellular ATP. PH-NQ and PH-MeHQ at a concentration of more than 0.1 g/L already showed an inhibitory effect on the luciferase reaction. In general, it was found that HC have a similar effect on both luciferases (bacterial and firefly) used in bioluminescent analysis methods based on the oxidation of their substrates. In addition to the relationships already described above, it is obvious that the minimum inhibitory effect was obtained in the case of the introduction of FA and its modified derivatives into the systems.

The analysis of the determination of the intracellular ATP concentration by the bioluminescent luciferin-luciferase method showed that the inhibitory effect of PH-NQ and PH-MeHQ substances was much stronger in comparison with FA-HQ preparations. The difference in the effect of HC on cells included in microbial consortia is primarily associated with differences in their chemical structure. It is known that the structure of FA molecules is characterized by the presence of a large number of aliphatic groups, which gives them hydrophilic properties, while HA is characterized by greater hydrophobicity due to the presence of aromatic groups in the structure, which apparently give HA a greater inhibitory effect against acetoclastic methanogens [37].

When studying the efficiency of biogas accumulation and its composition under the action of natural anaerobic consortia in the presence of different HC variants, it was shown that the use of FA derivatives chemically modified with hydroquinone residues did not lead to a decrease in the efficiency of methanogenesis and in some cases even stimulated the formation of biogas. This may be due to the fact that cells of microorganisms that are part of anaerobic consortia are able to use FA as additional substrates, similarly to what has been previously shown in other studies [16,38].

In the case of the use of HA preparations modified with quinone derivatives, a decrease in the efficiency of methanogenesis and the level of methane accumulation under the influence of various anaerobic consortia with an increase in the initial concentration of HC in nutrient media was observed. It should be noted that, using the example of various methanogenic consortia, other researchers have shown that partial inhibition of methanogenesis (by 40%) is possible at a HC concentration of 10 g/L and complete inhibition of CH_4_ formation only at 20 g/L HC [12]. Other researchers achieved the same effect at significantly higher HC concentrations.

In known study, a 35% reduction in biogas production was observed when cultivation of anaerobic sludge was undertaken in the presence of HC at a concentration of 10–20% HS (Humic substances)/VSS (Volatile suspended solids (mg/L)). At the same time, the portion of CH_4_ in the resulting biogas decreased by only 10–15% in the same studies [13]. Under conditions of a semi-continuous process of methanogenesis, an increase in the concentration of HC in the medium up to 20 HS/VSS also led to a decrease in the formation of biogas by 56% and the average fraction of CH_4_ in it—by 6.8% [14]. In one another study, a decrease in CH_4_ synthesis from 337.7 ± 1.5 mL CH_4_/g VS (volatile solids) to 144.3 ± 0.7 mL CH_4_/g VS was noted in samples of anaerobic sludge when humic acid sodium salt (AR, Aladdin) was added to the medium at a concentration of 0.6 g HM (humic matter)/g VS [15]. Thus, these results confirm that HC does indeed inhibit energy conversion efficiency in anaerobic digestion systems.Comparing the known results with the data obtained in this work, it can be argued that by chemical modification of HC, based on the use of knowledge about the reduction of methane emission under natural conditions (swamps), it is possible to obtain samples of HC derivatives, the use of which makes it possible to influence the composition of biogas formed under the action of anaerobic consortia, towards a significant decrease in the proportion of CH_4_ (in some cases, up to 100%). Such an approach can be taken into account to suppress the emission of landfill gases in real landfills and solid waste dumps. As it turned out, the inhibitory effects of modified HA preparations on the process of methanogenesis in the case of using immobilized cells were less pronounced in comparison with free cells.

It is known that an important characteristic of immobilized cells that significantly distinguishes them from suspension cells is their high operational stability [39]. In this regard, in order to suppress the metabolic activity of immobilized cells, it is likely that slightly higher concentrations of HC will be required than for suspension cells or re-introduction of HC derivatives in the case of their practical application in landfills to suppress methanogenesis. This result of observations should be necessary taken into account for the further work with landfills problems.

Thus, in this work, it was possible to significantly increase the ability of HC by their chemical modification with NQ and MeHQ to act as TAEs in anaerobic systems and significantly reduce the amount of biogas produced and the proportion of methane in it by introducing these synthetic HC derivatives into the media for the cultivation of methanogenic consortia in free or immobilized form.

## 5. Conclusions

As a result of the studies carried out, HA preparations were obtained, modified with NQ and MeHQ, characterized by a high electron-acceptor ability, the introduction of which into the environment leads to a decrease in the energy status and metabolic activity of cells of microorganisms present in anaerobic natural methanogenic consortia. In addition to reducing the efficiency of methanogenesis, it was also possible to change the composition of the resulting biogas towards a significant decrease in the proportion of CH_4_, including for the first time to show that such a decrease can reach almost 100% under certain conditions. The inhibitory effects of modified HA preparations in relation to methanogenesis were less pronounced if the anaerobic cells were immobilized. It was found that both bacterial and firefly luciferase can be used to assess the effect of different HC on various cells of microorganisms. The obtained results constitute important information that can be taken into account in the further development of scientifically grounded approaches to effective and directionally controlled suppression of gas emissions at real landfills and solid waste dumps.

## Figures and Tables

**Table 1:** Redox capacity of natural and quinone-enriched HC.

Sample	PH	FA	FA-HQ	PH-MeHQ	PH-NQ
Redox capacity (mmol/g)	0.30 ± 0.05	1.90 ± 0.20	0.90 ± 0.10	0.20 ± 0.05	0.10 ± 0.05

**PH:** potassium humate. **FA**: fulvic acids. **FA-HQ**: fulvic acids after modification with hydroquinone. **PH-MeHQ**: potassium humate after modification with 2-methylhydroquinone. **PH-NQ**: potassium humate after modification with 2-hydroxy-1,4-naphtoquinone.

## Supplementary Materials

The following are available online at https://www.mdpi.com/2076-3921/9/11/1140/s1, Table S1: *p*-values of pairwise multiple comparisons (Holm-Sidak method) after one-way ANOVA of the residual bioluminescence intensity of immobilized *P. phosphoreum* B-1717 cells with different various HC concentration. n.s.—statistically not significant (i.e., *p* > 0.05).
