# Supplementary material for: Suppression of Methane Generation during Methanogenesis by Chemically Modified Humic Compounds
Source: Antioxidants (Basel). 2020 Nov 17;9(11):1140. doi: 10.3390/antiox9111140 (PMC7698265; doi:10.3390/antiox9111140)
Supplement: Supplementary file 1 [file antioxidants-09-01140-s001.pdf]

Table S1. Table S1. p-values of pairwise multiple comparisons (Holm-Sidak method) after one-way ANOVA of the residual bioluminescence intensity of immobilized *P. phosphoreum B-1717* cells with different various HC concentration. n.s. – statistically not significant (i.e. p>0.05).

HC	Concentration, g/L	0	0.1	0.5	1	5
PH	0.1	<0.001 (t=9.836)				
	0.5	<0.001 (t=20.830)	<0.001 (t=10.993)			
	1	<0.001 (t=45.131)	<0.001 (t=35.295)	<0.001 (t=24.301)		
	5	<0.001 (t=52.653)	<0.001 (t=42.817)	<0.001 (t=31.835)	<0.001 (t=7.522)	
	10	<0.001 (t=57.629)	<0.001 (t=47.793)	<0.001 (t=36.799)	<0.001 (t=12.498)	<0.001 (t=4.976)
FA	0.1	n.s				
	0.5	0.002 (t=4.041)	n.s			
	1	<0.001 (t=10.440)	<0.001 (t=8.588)	<0.001 (t=6.399)		
	5	<0.001 (t=14.145)	<0.001 (t=12.292)	<0.001 (t=10.103)	0.003 (t=3.705)	
	10	<0.001 (t=15.963)	<0.001 (t=14.111)	<0.001 (t=11.922)	<0.001 (t=5.553)	0.003 (t=3.656)
FA-HQ	0.1	n.s				
	0.5	<0.001 (t=7.787)	0.004 (t=3.179)			
	1	<0.001 (t=16.466)	<0.001 (t=13.858)	<0.001 (t=10.679)		
	5	<0.001 (t=24.065)	<0.001 (t=18.279)	<0.001 (t=18.279)	<0.001 (t=7.600)	
	10	<0.001 (t=27.612)	<0.001 (t=25.004)	<0.001 (t=21.825)	<0.001 (t=11.146)	0.002 (t=3.546)
PH-MeHQ	0.1	0.002 (t=3.633)				
	0.5	0.002 (t=6.778)	0.005 (t=3.115)			
	1	<0.001 (t=12.089)	<0.001 (t=8.426)	<0.001 (t=5.311)		
	5	<0.001 (t=15.498)	<0.001 (t=11.835)	<0.001 (t=8.720)	0.003 (t=3.409)	
	10	<0.001 (t=16.806)	<0.001 (t=13.143)	<0.001 (t=10.028)	<0.001 (t=4.717)	n.s
PH-NQ	0.1	0.002 (t=3.688)				
	0.5	<0.001 (t=6.788)	0.005 (t=3.119)			
	1	<0.001 (t=12.106)	<0.001 (t=8.437)	<0.001 (t=5.318)		
	5	<0.001 (t=15.484)	<0.001 (t=11.816)	<0.001 (t=8.697)	0.003 (t=3.379)	
	10	<0.001 (t=16.830)	<0.001 (t=13.162)	<0.001 (t=10.042)	<0.001 (t=4.724)	n.s
